# A quantitative split firefly luciferase complementation assay (SplitLUC) for in *planta* protein-protein interactions

**DOI:** 10.1007/s00709-025-02146-x

**Published:** 2025-12-11

**Authors:** Qianwei Liu, Rainer Kembügler, Francesc Felipe, Jathish Ponnu

**Affiliations:** 1 Joseph Gottlieb Kölreuter Institute for Plant Sciences (JKIP), Karlsruhe Institute of Technology (KIT), 76131 Karlsruhe, Germany; 2 Berthold Technologies GmbH & Co. KG, Calmbacher Straße 22, 75323 Bad Wildbad, Germany

**Keywords:** Protein–protein interactions, Split firefly luciferase complementation assay, Cooled charge-coupled device, NightSHADE, SplitLUC, CCD

## Abstract

Understanding protein-protein interactions (PPIs) *in planta* is essential for deciphering the molecular mechanisms underlying plant development and responses to environmental stresses. Here, we demonstrate the application of the split firefly luciferase complementation assay (SplitLUC) using a cooled charge-coupled device (CCD)-based plant imaging system and a microplate reader to detect and quantify PPIs *in planta*. As an example, we investigated the previously reported interaction between DET1- and DDB1-ASSOCIATED 1 (DDA1), a component of the CULLIN4 (CUL4)-E3 ubiquitin ligase complex, and PYR1-like 8 (PYL8), a known substrate of the same complex. Co-infiltration of Agrobacterium strains carrying DDA1-nLUC and cLUC-PYL8 constructs resulted in a robust luminescent signal upon addition of D-luciferin, which was visualised and quantified using the NightSHADE evo Plant Imaging System. Control combinations lacking either fusion partner or containing only empty vectors did not produce detectable luminescence, confirming the specificity of the interaction. To account for infiltration efficiency and variability in transgene expression, the luminescence values were normalised against fluorescence from co-infiltrated TagRFP, measured using a Tecan Spark microplate reader. This normalisation strategy effectively mitigated leaf-to-leaf variation in luminescence signals and demonstrated that the SplitLUC assay, when combined with fluorescence-based normalisation, provides a robust and reliable quantitative method for studying PPIs *in planta*. We propose that this approach is well-suited for investigating weaker interactions, assessing the influence of additional (bridge) proteins, and mapping interaction domains within the proteins of interest.

**Supplementary Information:**

The online version contains supplementary material available at 10.1007/s00709-025-02146-x.

## Introduction

Studying the physical interactions between proteins is crucial for understanding biological phenomena at the molecular level (Xing et al. 2016). In plants, protein-protein interactions (PPIs) and the dynamic assembly of protein complexes play essential roles in biochemical, physiological, and developmental processes, particularly in response to environmental constraints (Cuadrado and Van Damme 2024). Although *ex planta* or surrogate methods, such as the yeast two-hybrid (Y2H), are widely used for both individual and high-throughput PPIs (Brückner et al. 2009; Xing et al. 2016; Trimborn et al. 2022), they often lack the contextual specificity of plant tissues, developmental stages, and physiological conditions. Consequently, *in planta* approaches that offer better sensitivity and physiological relevance are preferred for capturing biologically meaningful protein associations.

Among the *in planta* techniques, co-immunoprecipitation (Co-IP) and mass spectrometry-based high-throughput assays, such as tandem affinity purification and proximity labelling, provide an overview of the composition of protein complexes (Lin and Lai 2017; Struk et al. 2018; Cuadrado and Van Damme 2024). While these methods can detect the co-existence of proteins within the same complex, they do not necessarily offer direct evidence of binary interactions between specific proteins of interest (POIs). More sophisticated *in planta* techniques, such as Förster Resonance Energy Transfer combined with Fluorescence Lifetime Imaging Microscopy (FRET-FLIM), enable the detection of direct physical interactions, including ternary complexes with scaffold or bridge proteins (Bücherl et al. 2014; Ponnu et al. 2019; Glöckner et al. 2022; Eljebbawi et al. 2025). Nevertheless, these methods often require expensive instrumentation and expertise, which can limit their accessibility.

In this context, there is a growing need for PPI assays that are both experimentally accessible and biologically reliable. One such approach involves splitting a reporter protein into two non-functional fragments, each fused to a POI. Upon interaction of the target proteins, the reporter fragments are brought into proximity as a functional unit, enabling detection. This principle underlies several widely used assays, including Bimolecular Fluorescence Complementation (BiFC) and the split-ubiquitin systems (Fetchko and Stagljar 2004; Stolpe et al. 2005; Grefen and Blatt 2012), the latter being particularly suited for membrane proteins. While BiFC is popular, it demands careful experimental design and extensive controls to reduce the false positives due to the persistent and often irreversible nature of fluorophore reconstitution (Kudla and Bock 2016).

**Fig. 1:**
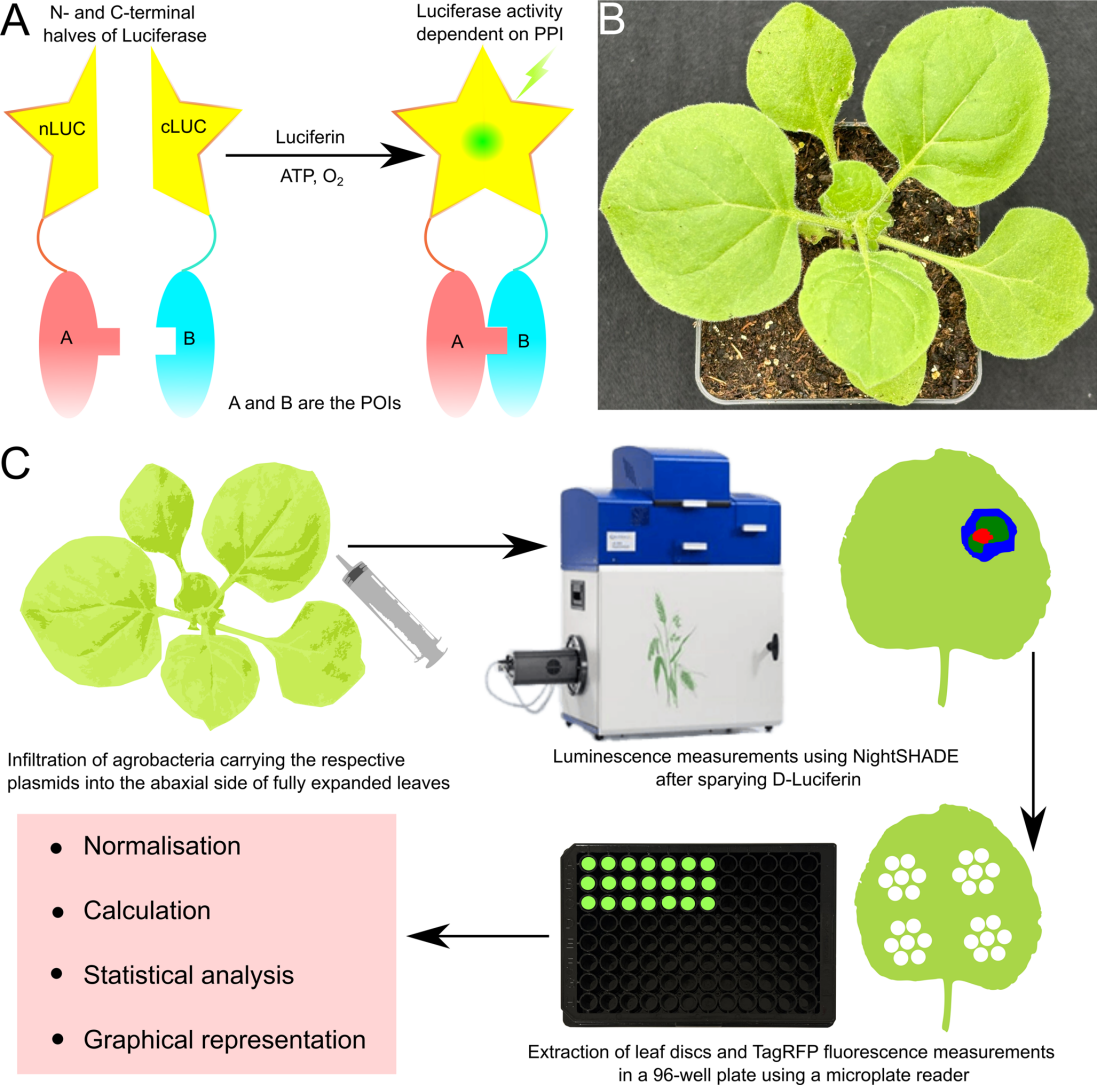
**SplitLUC assay for PPIs in ***Nicotiana benthamiana* (Nb) using NightSHADE (**A**). Schematic showing the principle of the SplitLUC assay. The POIs A and B are fused with nLUC and cLUC, respectively. Upon a physical interaction of **A **and **B**, the function of the full-length luciferase enzyme is reconstituted, resulting in the production of luminescence in the presence of the substrate luciferin. (**B**) Four-week-old Nb plant suitable for infiltration. (**C**) Workflow showing SplitLUC assay using NightSHADE. Abbreviations: nLUC, N-terminal fragment of luciferase enzyme; cLUC, C-terminal fragment of luciferase enzyme; POIs, proteins of interest; SplitLUC, split firefly luciferase complementation assay

A promising alternative for detecting PPIs *in planta* is the split firefly luciferase complementation assay (SplitLUC) (Chen et al. 2008), which utilises the N- and C-terminal halves of the luciferase enzyme (nLuc and cLuc, respectively; Fig. 1A). Several variations of this technique exist, including high-throughput formats using mammalian cell lines followed by luminescence measurements via plate readers. A widely adopted *in planta* method employs the *Nicotiana benthamiana* (Nb) transient expression system (Fig. 1B and C) (Chen et al. 2008; Gehl et al. 2011). In this approach, the POIs are fused to either nLuc or cLuc and transiently expressed in four-week-old Nb plants via agrobacterium-mediated infiltration (Kapila et al. 1997; Chincinska 2021). Upon interaction of the POIs, the luciferase fragments functionally reconstitute to form the luciferase enzyme capable of oxidising the substrate luciferin, resulting in the emission of luminescence (Fig. 1A). This signal can be detected and quantified using a cooled charge-coupled device (CCD) camera-based imaging system, such as the NightSHADE evo (Berthold Technologies) (Fig. 1C), or with a standard luminometer.

Here, we present an example of PPI between DET1-, DDB1-ASSOCIATED 1 (DDA1) and PYR1-like 8 (PYL8), two proteins from *Arabidopsis thaliana* (Irigoyen et al. 2014). DDA1 functions as a substrate adaptor within the CULLIN4 (CUL4)-based E3 ubiquitin ligase complex. PYL8, an abscisic acid receptor, serves as a substrate of CUL4-E3 and is targeted for ubiquitination and subsequent proteasomal degradation (Irigoyen et al. 2014; Nassrallah et al. 2018). The interaction between DDA1 and PYL8 has been previously demonstrated through Y2H, BiFC and Co-IP assays (Irigoyen et al. 2014). We utilised the CCD camera system of the NightSHADE evo to capture luminescence signals resulting from the interaction between DDA1-nLUC and cLUC-PYL8 and quantified the data using IndiGO™ software. To ensure consistent protein expression across all infiltration combinations, luminescence values were normalised to the expression of TagRFP, which was co-infiltrated alongside the Split-LUC constructs. The TagRFP fluorescence was measured using a Tecan Spark^®^ Microplate Reader. We demonstrate that our integrated method reliably detects and quantifies the interaction between DDA1 and PYL8 *in planta*, utilising the SplitLUC assay.

## Materials and methods

### Plasmid construction and transformation

**Fig. 2:**
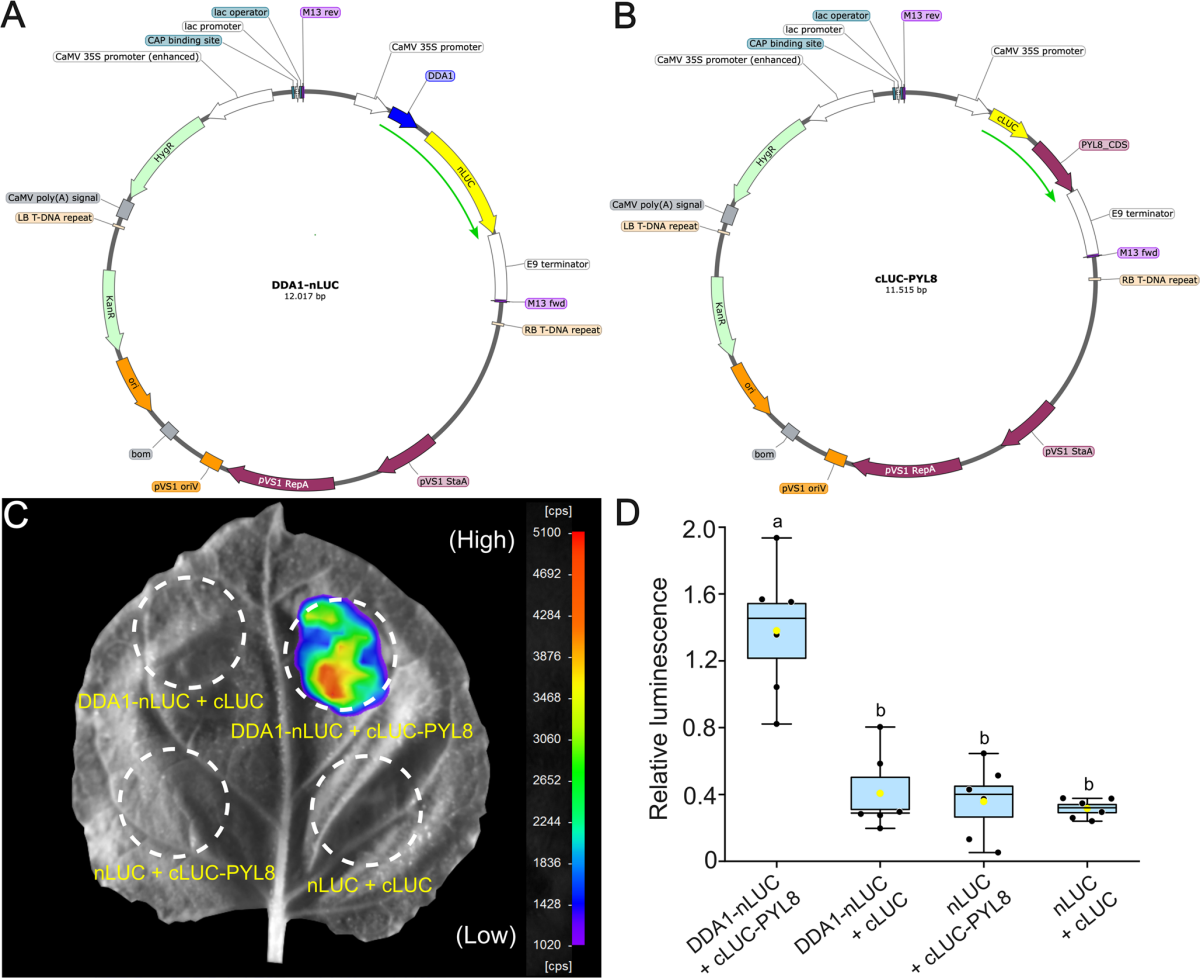
DDA1 physically interacts with PYL8 as demonstrated in the SplitLUC assay (**A**, **B**). Binary plasmids encoding DDA1-nLUC and cLUC-PYL8, respectively, are compatible with Agrobacterium transformation and subsequent expression in plants. The green arrow denotes the fusion fragments in-frame. The maps were made using SnapGene^®^ software. (**C**) Luminescence image superimposed on the white light image of the infiltrated representative Nb leaf, showing luminescence represented in false colours. Images were taken using the NightSHADE imaging system. The scale shows colours and the corresponding signals in counts per second (cps). (**D**) Box and whisker plot with data points (black circles), showing the relative luminescence (normalised to TagRFP fluorescence) from similarly aged leaves from 6 different plants (*n* = 6) infiltrated with n-LUC/c-LUC fusions along with TagRFP. The blue blocks represent the interquartile range. Whiskers represent the minimum and maximum. The average values are represented as yellow circles. Black dots denote individual data points. Black horizontal lines spanning the width of the boxes represent median values. Statistical analyses were conducted using one-way ANOVA, followed by Tukey’s post hoc test for multiple comparisons

DDA1 (AT5G41560) and PYL8 (AT5G53160) coding sequences were amplified from *Arabidopsis thaliana* cDNA using Q5 High-Fidelity DNA Polymerase (New England Biolabs), with the respective primer pairs listed in Table 1. The resulting PCR products were assembled into the pCAMBIA1300-nLUC and pCAMBIA1300-cLUC vectors (Chen et al. 2008) via Gibson assembly. Before assembly, the vectors were linearised by restriction digestion with KpnI and SalI, and the cloning was performed using the ClonExpress II One Step Cloning Kit (Vazyme), following the manufacturer’s protocol. The resulting recombinant plasmids are illustrated in Fig. 2A and B. These constructs were subsequently introduced into *Agrobacterium tumefaciens* strain GV3101(pMP90) via heat shock transformation. The potential transformed colonies were screened by PCR to confirm the presence of the respective plasmids.

**Table 1:** Primers used in this study

Primer name	Sequence
pJP448_FP	ggacgagctcggtacatggcgtcgattctgggt
pJP448_RP	gcgtacgagatctggcgtaaaccctgagtagatgaagaagaagacgcagc
pJP452_FP	gtcccggggcggtacGATGGAAGCTAACGGGATTGAGAACT
pJP452_RP	cgaaagctctgcaggTTAGACTCTCGATTCTGTCGTGTCTTGAAC

## Agrobacterium transformation and culture preparation

Transgenic Agrobacterium strains harbouring gene fragments encoding the POIs fused to either nLUC or cLUC (Fig. 2A) are cultured individually in 20 ml of LB medium supplemented with appropriate antibiotics. Cultures are grown in 50 ml conical flasks at 30 °C with shaking (approximately 200 rpm) for 24 h. Following incubation, bacterial cells are harvested by centrifugation at 4000 rpm for 15 min at room temperature. The supernatant is discarded, and the resulting pellet is resuspended in 1 ml of Agromix buffer (10 mM MgCl₂·6 H₂O, 10 mM MES, pH 5.6), freshly supplemented with acetosyringone (3,5-dimethoxy-4-hydroxyacetophenone) at a final concentration of 3 mg/ml. The optical density of each bacterial suspension is adjusted to OD _600_ = 0.8. Equal volumes of the nLUC- and cLUC-fusion cultures, along with Agrobacterium carrying the TagRFP construct (Schwenk et al. 2021), are mixed for co-infiltration (see Fig. 2C; Table 2). The mixture is incubated in the dark at room temperature for 30 min before infiltration.

**Table 2:** Plasmids used in this study

Glycerol stock	Full name	Remarks
gl-0392	pJP448_pCAMBIA1300-nLUC_DDA1	DDA1-nLUC
gl-0393	pJP452_PCAMBIA1300-cLUC_PYL8	cLUC-PYL8
gl-0466	pCAMBIA1300-nLUC	nLUC; Chen et al., 2008
gl-0465	pCAMBIA1300-cLUC	cLUC; Chen et al., 2008
gl-0496	4685_pCHF230_35S_TagRFP	TagRFP; Schwenk et al., 2021

## Plant infiltration and incubation

Healthy 4-week-old Nb plants are selected for infiltration. Fully expanded leaves from the top 3–4 leaves of each plant are preferred. The bacterial suspension is infiltrated into the abaxial side of the leaves using a needleless syringe, targeting four distinct zones per leaf (Fig. 2C). Post-infiltration, the plants are incubated in a dark, humid environment for 24 h to facilitate bacterial uptake and initial expression. Subsequently, plants are transferred to long-day growth conditions (16 h light/8 h dark) for an additional 48 h to allow for optimal protein expression.

## Luminescence and fluorescence measurements

For luminescence imaging, infiltrated leaves were detached and placed abaxial side up on MS-agar plates. Each plate is sprayed with 10 ml of D-luciferin working solution, prepared as a 5 mM solution of D-luciferin potassium salt (Synchem) containing 0.025% Triton X-100 in distilled water. Spraying is performed using a 50 ml spray bottle to ensure even distribution. The leaves are then incubated in the dark for 15 min to quench chlorophyll autofluorescence (Maxwell and Johnson, 2000) before imaging. Infiltrated leaves of similar developmental stage were selected from six independent plants (*n* = 6) for luminescence and fluorescence measurements.

 Luminescence was detected using the NightSHADE evo In Vivo Plant Imaging System integrated with IndiGO™ software (Berthold Technologies, Germany). Alternatively, any CCD-camera-based luminescence detection can be employed. Signal acquisition parameters in the IndiGO™ software were set as follows: 20-second exposure time, low gain, slow readout speed, and 8 × 8 binning. Post-acquisition processing included cosmic ray suppression and background correction to enhance signal fidelity. Following luminescence detection, regions exhibiting luciferase activity were identified based on the imaging data and marked accordingly. After luminescence detection, the obtained images are visualised using indiGO™ software, which also allows the extraction of quantitative information to a Microsoft Excel^®^ file. When using alternative CCD camera systems that lack integrated software, the obtained images can be analysed using the freely available Fiji software (https://imagej.net/software/fiji/downloads) to extract quantitative information.

Leaf discs corresponding to these regions were excised using a leaf punch to fit the wells of a 96-well half-area black microplate (Greiner). Discs were floated abaxial side up in 200 µl of distilled water per well. Fluorescence measurements were conducted using a Tecan Spark^®^ microplate reader (Tecan Group). Any plate reader that can measure fluorescence in a 96-well format is acceptable as an alternative. TagRFP was excited at 543 nm, and emission was recorded at 589 nm. The gain was set to optimal, and the number of flashes per well was set to 20 to ensure robust signal acquisition. Luminescence values were normalised to TagRFP fluorescence (Luminescence value (cps) from the ROI/mean RFP fluorescence (cps) from leaf discs extracted from the ROI) to calculate relative luminescence units (RLUs). These normalised values were subjected to statistical analysis using OriginPro^®^ software. Group comparisons were performed using one-way ANOVA followed by Tukey’s post hoc test to determine statistical significance.

## Results and discussion

Figure 2C illustrates the luminescence observed from various combinations of transgenic agrobacteria infiltrated into Nb leaf sections. Upon the addition of D-luciferin, the luminescence signals measured as counts per second (cps) were detected specifically in leaf areas co-infiltrated with DDA1- and PYL8-fusion constructs, as visualised using the NightSHADE imaging system. The luminescence image was superimposed on the leaf image under white light to indicate the infiltrated regions and the origin of the signal. The regions corresponding to the infiltrated areas were premarked on the adaxial side of the leaves. Additionally, these areas were readily recognisable at the macroscopic level due to visible changes in leaf tissue, independent of the luminescence signal. A strong luminescence signal was observed in the DDA1-nLUC and cLUC-PYL8 co-infiltrated samples (Fig. 2C). In contrast, the empty controls or nLUC- or cLUC-fusion proteins alone (Fig. 2C) did not produce any detectable luminescence. This confirms that the specific interaction between DDA1 and PYL8 *in planta* reconstituted the functional luciferase enzyme (shown as a schematic in Fig. 1A) and mediated the enzymatic conversion of D-luciferin into a luminescent signal, which was captured by the NightSHADE system (Fig. 2C).

Quantitative data from similar images were extracted from defined regions of interest (ROI) using the IndiGO™ software. To further quantify the interaction, leaf discs corresponding to the ROIs (5–6 leaf discs per combination, as shown in Fig. 1C as a schematic) were excised using a leaf punch and their tagRFP fluorescence was measured using a Tecan Spark^®^ plate reader. Relative luminescence values were calculated by normalising luminescence to tagRFP fluorescence to obtain the relative luminescence (Fig. 2D and S1). Data from six independently infiltrated, similarly aged leaves of six different plants were used to generate the graph in Fig. 2D. The DDA1-nLUC and cLUC-PYL8 co-infiltrated samples showed significantly high relative luminescence, indicating a specific and robust interaction between DDA1 and PYL8 proteins *in planta*, which was confirmed in an earlier study using Y2H, BiFC and Co-IP (Irigoyen et al. 2014).

Although SplitLUC assays have been widely used to study PPIs *in planta*, many studies rely primarily on qualitative imaging of luminescence, without incorporating quantitative measurements. While this approach may be sufficient for detecting strong interactions, where the POIs produce stronger luminescence than controls, it often fails to capture subtle differences in interaction strengths. This limitation becomes particularly important when mapping interaction domains within the POIs or evaluating the influence of a third protein on the interaction between two proteins. In such scenarios, leaf-to-leaf variation in luminescence signals can significantly affect data interpretation. These variations may arise from a range of biological and technical factors, including differences in plant and leaf age, infiltration volume, and timing (Bashandy et al. 2015). Even with careful selection of morphologically and developmentally similar plants, inconsistencies in infiltration efficiency, transgene expression, and subsequent protein production can introduce bias. To ensure reliable and reproducible quantitative data across experimental replicates and time points, it is essential to normalise these variables.

Previous efforts to address these limitations include the floated-leaf luciferase complementation assay, in which luminescence signals were normalised against GUS activity or co-expressed fluorescent proteins such as GFP or FP611, followed by microscopy and immunoblotting to confirm uniform expression levels of nLUC- and cLUC-tagged POIs (Gehl et al. 2011). Building further upon these strategies, we simplified the workflow in a dual-readout approach by integrating luminescence imaging using the NightSHADE with fluorescence quantification from leaf discs. Even though the variation of TagRFP fluorescence among the individual leaf discs within each combination was evident (Supplemental Fig. 1), the differences among the combinations are not significantly different, indicating statistical robustness and sample sufficiency in our method. Based on our experience with this system, we recommend using at least 6 to 7 leaf discs (assuming the infiltration area is less than 2.5 cm in diameter, and a leaf punch of 8 mm diameter is used), each derived from six independent leaves, ideally from six different plants, to obtain reliable data in SplitLUC quantitative assays.

## Conclusions

We have developed and standardised a quantitative splitLUC assay for *in planta* PPIs, integrating luminescence measurements and normalising them with the fluorescence to reduce leaf-to-leaf variations in protein expression. Unlike the traditional image-based SplitLUC systems, our approach offers a rapid and reproducible quantitative method, particularly useful in assessing interaction strengths without the need for further biochemical validations. Although alternative platforms exist for luminescence quantification, NightSHADE stands out due to its ease of use, visual clarity, and has a distinct advantage in capturing high-resolution luminescence signals intuitively. The integrated IndiGO™ software further streamlines the analysis of spatially resolved data by allowing efficient extraction of signal intensities from ROIs. This is beneficial while quantifying luminescence across heterogeneous leaf sectors in the infiltration zone, avoiding the need for manual segmentation, which is time-consuming and error-prone.

For detecting strong and specific PPIs via the SplitLUC assay, NightSHADE-based visualisation and quantification alone may produce robust data. However, while assaying weaker interactions and quantifying interaction strengths among truncated versions of proteins or ternary complexes, further normalisation using fluorescence values will be crucial in compensating for variability. We believe that our assay holds potential for applications involving bridge proteins that modulate protein-protein interactions, as recently demonstrated (Trimborn et al. 2025), thereby extending its utility for dissecting complex interaction networks *in planta*.

## Supplementary Information

Below is the link to the electronic supplementary material.

Supplementary Material 1 (PDF 233 KB)

## Data Availability

No datasets were generated or analysed during the current study.
