# Supplementary material for: A quantitative split firefly luciferase complementation assay (SplitLUC) for in planta protein-protein interactions
Source: Protoplasma. 2025 Dec 11;263(3):1067–73. doi: 10.1007/s00709-025-02146-x (PMC13109126; doi:10.1007/s00709-025-02146-x)
Supplement: Supplementary file 1 — Supplementary Material 1 (PDF 233 KB) [file 709_2025_2146_MOESM1_ESM.pdf]

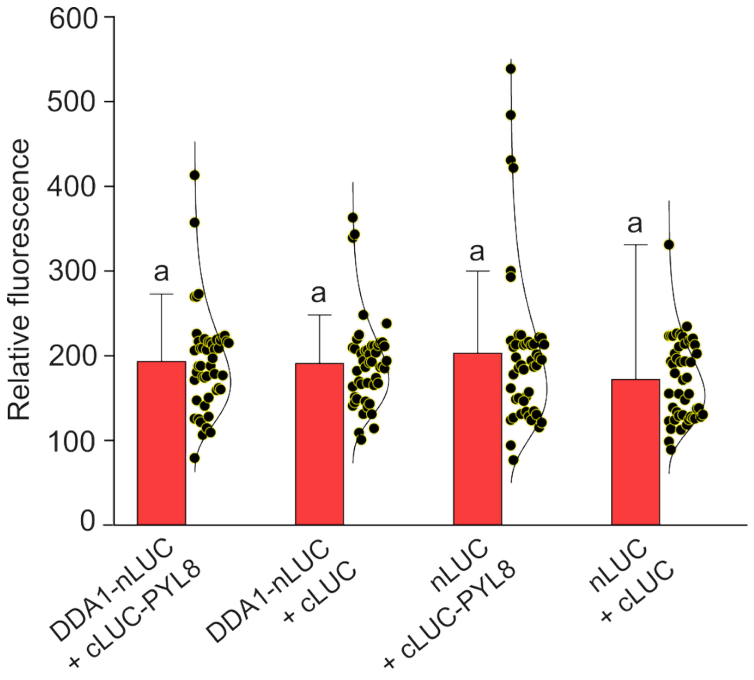

Supplemental Figure 1: Relative fluorescence from the leaf discs co-expressing TagRFP

Relative fluorescence was measured in *Nicotiana benthamiana* leaf discs co-expressing TagRFP and SplitLUC fusion proteins, compared to non-infiltrated leaf discs. For each combination, more than 40 leaf discs (n > 40) were analysed, collected from six independent plants. Statistical analysis was performed using one-way ANOVA followed by Tukey's multiple comparison test. Bars represent the mean fluorescence values, with individual data points shown adjacent to each bar. Error bars indicate the interquartile range.
